# Supplementary material for: The causal relationship between the human gut microbiota and pyogenic arthritis: a Mendelian randomization study
Source: Front Cell Infect Microbiol. 2024 Nov 26;14:1452480. doi: 10.3389/fcimb.2024.1452480 (PMC11629706; doi:10.3389/fcimb.2024.1452480)
Supplement: Supplementary file 1 [file DataSheet1.zip › mendelian test/ebi-a-GCST90027826.csv_forest.pdf]

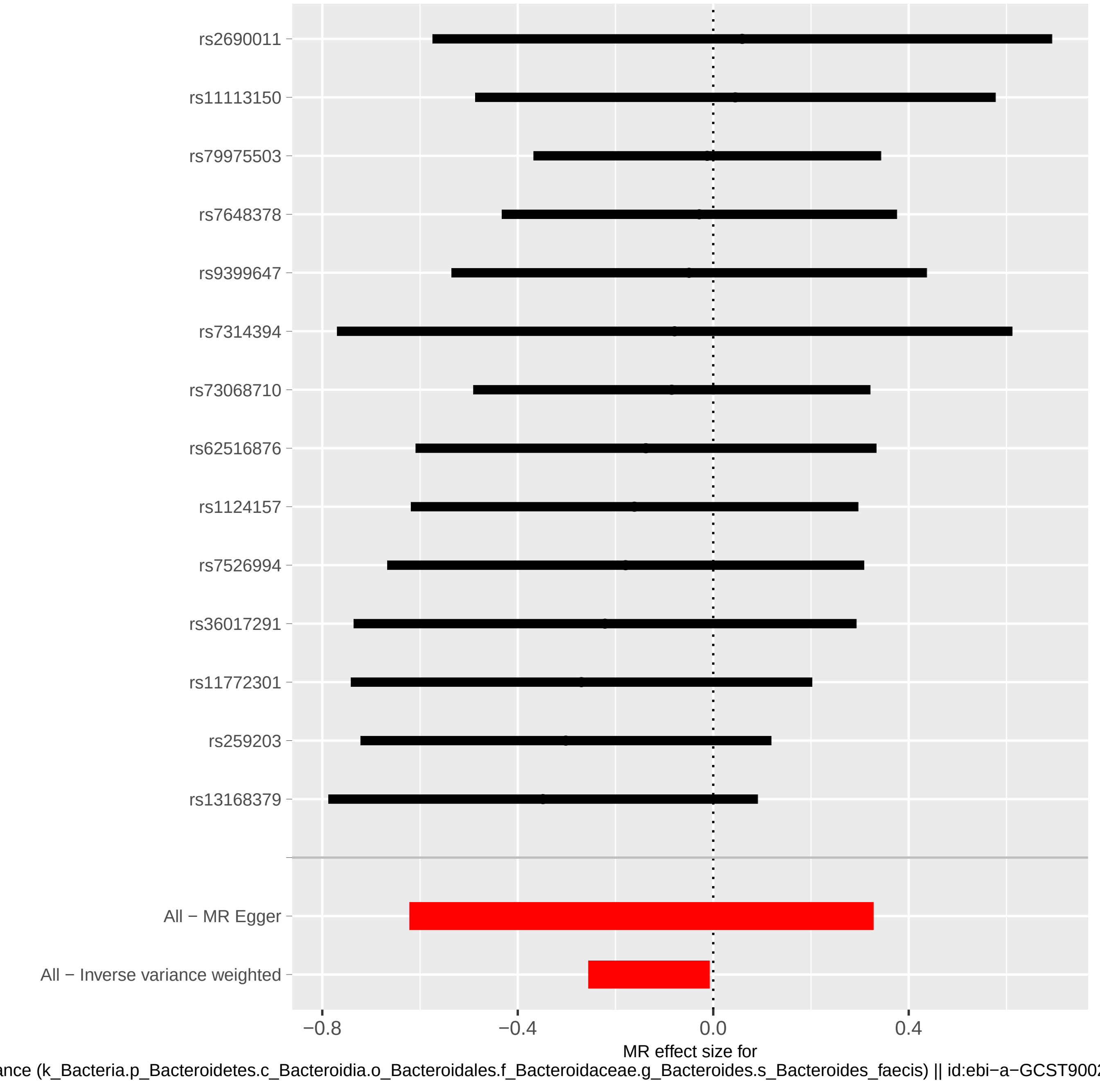
rs2690011
rs11113150
rs79975503
rs7648378
rs9399647
rs7314394
rs73068710
rs62516876
rs1124157
rs7526994
rs36017291
rs11772301
rs259203
rs13168379
All − MR Egger
All − Inverse variance weighted
−0.8
−0.4
0.0
0.4
MR effect size for
ance (k_Bacteria.p_Bacteroidetes.c_Bacteroidia.o_Bacteroidales.f_Bacteroidaceae.g_Bacteroides.s_Bacteroides_faecis) || id:ebi−a−GCST900
